# Research Progress and Future Trends of Microfluidic Paper-Based Analytical Devices in In-Vitro Diagnosis

**DOI:** 10.3390/bios12070485

**Published:** 2022-07-03

**Authors:** Taiyi Zhang, Feng Ding, Yujing Yang, Gaozhen Zhao, Chuanhao Zhang, Ruiming Wang, Xiaowen Huang

**Affiliations:** State Key Laboratory of Biobased Material and Green Papermaking, School of Bioengineering, Qilu University of Technology, Shandong Academy of Sciences, Jinan 250353, China; zty2020bio@126.com (T.Z.); bio_ding@126.com (F.D.); 10431211246@stu.qlu.edu.cn (Y.Y.); 10431210986@stu.qlu.edu.cn (G.Z.); 10431211102@stu.qlu.edu.cn (C.Z.); ruiming3k@163.com (R.W.)

**Keywords:** in vitro diagnosis, paper-based microfluidics, colorimetric analysis, fluorescence, chemiluminescence, electrochemical signal

## Abstract

In vitro diagnosis (IVD) has become a hot topic in laboratory research and achievement transformation. However, due to the high cost, and time-consuming and complex operation of traditional technologies, some new technologies are being introduced into IVD, to solve the existing problems. As a result, IVD has begun to develop toward point-of-care testing (POCT), a subdivision field of IVD. The pandemic has made governments and health institutions realize the urgency of accelerating the development of POCT. Microfluidic paper-based analytical devices (μPADs), a low-cost, high-efficiency, and easy-to-operate detection platform, have played a significant role in advancing the development of IVD. μPADs are composed of paper as the core material, certain unique substances as reagents for processing the paper, and sensing devices, as auxiliary equipment. The published reviews on the same topic lack a comprehensive and systematic introduction to μPAD classification and research progress in IVD segmentation. In this paper, we first briefly introduce the origin of μPADs and their role in promoting IVD, in the introduction section. Then, processing and detection methods for μPADs are summarized, and the innovative achievements of μPADs in IVD are reviewed. Finally, we discuss and prospect the upgrade and improvement directions of μPADs, in terms of portability, sensitivity, and automation, to help researchers clarify the progress and overcome the difficulties in subsequent μPAD research.

## 1. Introduction

Microfluidic technology is a technique for the precise control and manipulation of microscale fluids. A microfluidic device is a platform to realize this technology. Researchers have studied and applied microfluidic devices in medicine [1,2,3,4], food safety [5], and environmental monitoring [6].

The core component of microfluidic devices is microfluidic chips. Traditional microfluidic chips are based on glass, a silicon wafer, polymer, and control fluid, using microscopic fluid properties. Paper-based microfluidic chips are mainly made of paper fiber, and control the fluid using capillary force. Traditional microfluidic chips have some advantages that paper-based microfluidic chips cannot replicate. For example, some microscopic fluid properties exploited in traditional microfluidic chips are not available on paper-based chips. Of course, the application of traditional microfluidic chips is not facile, due to their disadvantages, such as complex operation, poor flexibility, and high requirements for the manufacturing environment. However, paper-based microfluidic chips can overcome these disadvantages [7].

**Figure 1:**
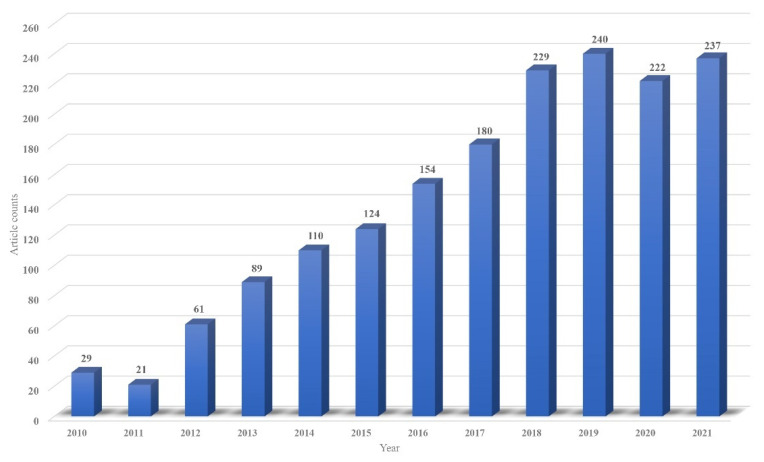
The bar chart shows the number of papers published each year from 2010 to 2021. These data are from the Web of Science, and the retrieved content is “paper-based microfluidic device (Topic)”.

μPADs are developed from paper-based microfluidic chips. Owing to their advantages, μPADs have gradually attracted the interest of researchers [8,9], and the annual number of related papers in the past 12 years is shown in Figure 1. They use specific materials to draw the graphics of functional units and channels on the paper substrate, to realize the directional flow of liquid in the hydrophilic channels with the help of the capillary action of a cellulose skeleton. Paper is not only cheap and easy to manipulate but also has better biocompatibility and provides a good background contrast for colorimetric reactions. The prototype of paper-based microfluidic chips can be traced back to the 1940s, when Muller’s research group used paraffin to form channels on filter paper and observed the diffusion and separation of a pigment solution on paper. In addition, immunochromatography, which appeared in the 1980s, can also be regarded as one of the early paper-based microfluidic technologies. In most existing reviews, Whiteside’s research group at Harvard University [10] is recognized as the first team reporting paper-based microfluidic chips. Since their report, paper-based microfluidic chips and devices with different functions and processing methods have emerged. Whitesides’ research group combined paper chips with scanning devices to create a μPAD for quantitative analysis [11] and made a three-dimensional (3D) paper chip for the first time, by stacking multiple layers of paper chips with double-sided tape [12]. In 2009, Whitesides’ research group [13] and Lin’s research group [14] proposed using the wax printing method to fabricate paper-based microfluidic chips. Their method is widely used because of its simplicity and low cost. In the same year, Dungchai et al. produced a paper chip based on electrochemical signal detection, by first printing electrodes on filter paper [15]. This method significantly improves the detection sensitivity of paper-based microfluidic chips. In 2011, Liu et al. realized the fabrication of 3D paper chips using the folding method [16], which is more convenient and efficient than the fabrication method provided by Whitesides’ research group.

Admittedly, in their early stages, paper-based microfluidic chip detection limits (LODs) were not low enough, susceptible to contamination, and mechanically weak. To solve these problems, new nanomaterials, sealing devices, and advanced sensors are used in μPADs by researchers [17,18]. Among these, adding sensors is a research hotspot. Many research teams have explored the construction of fluorescence sensing [19], electrochemical sensing [20], and colorimetric sensing [21] on the paper chip platform. They then studied and designed a series of low-cost, convenient, and rapid μPADs [22,23,24,25,26,27], which promoted the application and development of paper-based microfluidic technology in biological and environmental analysis.

IVD refers to the analysis of samples from the human body, to obtain corresponding biochemical and molecular parameters for diagnosis and treatment. Traditional IVD technology generally requires the operation of complex and large analytical instruments, which hinders the popularization and application of this technology. Therefore, IVD began to develop towards POCT, a refined field. POCT is characterized by its fast detection speed, low cost, and simple operation. μPADs are growing rapidly in this field because they meet part of the demand of the POCT market [28,29].

With the increasing research on paper-based microfluidic chips in IVD, it is helpful to systematically understand the development situation and future trends in this field, by summarizing and categorizing its achievements. Previous reviews of paper-based microfluidic chips mainly focused on fabrication methods and there is a lack of reviews on the main applications of the devices in specific disease detection and diagnosis processes [24,30,31]. In this paper, we first briefly introduce the origin of μPADs and their role in promoting IVD, in the introduction section. Then, the processing and detection methods of μPADs are summarized, and the innovative achievements of μPADs in IVD are reviewed. Finally, we discuss and prospect the upgrade and improvement directions of μPADs, in terms of portability, sensitivity, and automation, to help researchers clarify the progress made and overcome difficulties in subsequent μPADs research.

## 2. Processing Methods of μPADs

The channel patterns of μPADs are generally completed by printing and modifying fluid boundaries drawn on paper. The formed channel controls liquid transmission in the devices and provides a location and conditions for biochemical reactions. μPADs were two-dimensional (2D) chips during their early development. There are two processing methods for 2D paper chips: the first method is direct hydrophilic treatment; the second is to conduct a comprehensive hydrophobic treatment, followed by a hydrophilic treatment. Researchers can create three-dimensional chips by superimposing modified paper chips [32].

**Figure 2:**
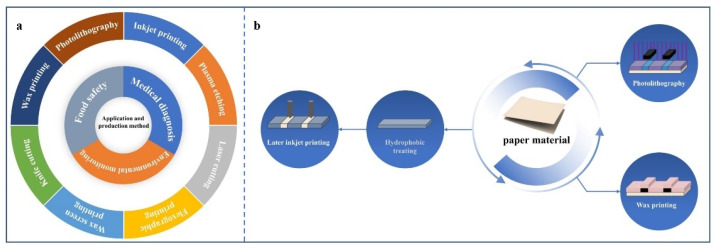
(**a**) Diagram showing the main application field (inner ring) and manufacturing methods (outer ring) of μPADs. (**b**) Schematic diagram of the three usual processing methods for paper-based microfluidic channels. Principle of photolithography: this melts the photoresist outside the mask into the inner pores of the paper material and forms a physical barrier after re-solidification. Wax printing principle: it is melted by heating the wax material, generating a physical barrier to the void of the paper material in the uncovered area of the mold. Inkjet printing principle: the hydrophilic material is printed on the paper, to form a specific channel after the paper material hydrophobic treatment.

At present, several methods, including photolithography [33], wax printing [13], inkjet printing [34], plasma etching [35], knife cutting [36], laser cutting [37], flexographic printing [38], and wax screen printing [39], have been developed for fabricating and modifying the channels of μPADs for varied applications (Figure 2a). Researchers used these methods to accomplish different purposes and to discover their advantages and disadvantages in practice (Table 1). Of these methods, the most commonly used methods are the first three (Figure 2b).

At the beginning of the development of paper-based microfluidic technology, photoresists were one of the primary materials for making channel boundaries. Its principle is to block the porous structure of the paper material physically. The photoresist used in μPADs is mainly the SU-8 photoresist, an epoxy, near-ultraviolet negative photoresist produced using microlithography chemicals. Martinez et al. used SU-8 photoresist-coated patterned paper to create millimeter-sized channels [10].

However, photolithography is complicated and costly. Thus, wax printing became a popular modification method. Although one group prepared a negative epoxy photoresist with SU-8, triaryl thio-hexafluorophosphate, and propylene glycol methyl ether acetate, which reduced the cost of preparing a μPAD by lithography, these manufacturing steps are too cumbersome and complex. As a hydrophobic material, wax can be physically deposited in paper materials, to form a barrier [40]. When the wax material is heated above the melting point, it will penetrate the gap of the paper fiber and form a three-dimensional hydrophobic barrier after solidification, to complete the production of the channel pattern. In some cases, to ensure the accuracy of channel pattern creation, the wax printing technology needs to adjust the heating time and temperature according to the melting point of the wax and the porosity and thickness of the paper materials.

Inkjet technology is also frequently used in the manufacture of μPADs, because of its high speed, precision, and automation [41]. However, it should be noted that the resolution of some printers may be problematic, because of the inkjet printer’s design when printing multiple layers. In addition, many solvents that are necessary to dissolve the sensing reagents may be volatile, which will lead to the printing of the wrong amount of reagent [42]. These shortcomings make the standardization benefits of inkjet printing inferior to flexographic printing.

The cutting method is becoming increasingly popular, because of its simple operation and lack of chemical reagents. From the initial handheld device [43] to the current laser cutting [44], the accuracy of the cutting method is improving with the progress of technology.

Some researchers have also attempted to combine certain production methods. Yu et al. combine inkjet printing and paper cutting to make paper devices. Experiments showed that the technique has excellent reproducibility and sensitivity [45].

## 3. μPADs Based on Different Detection Methods

The detection method used is particularly significant for the performance of μPADs. At present, there are more paper-based detection products using colorimetry and fluorescence, and fewer using chemiluminescence. However, researchers have developed many excellent μPADs using various detection methods in published papers. Representative research on detection sensitivity in μPADs is presented in Table 2. The immunocapture approach is frequently utilized in μPADs, because of its excellent specificity, and these example studies are no exception. Although some research has achieved low detection limits, sample processing techniques need to be improved because they are time-consuming. Next, we will discuss the detection methods used in μPADs.

### 3.1. μPADs Based on Colorimetric Analysis

**Figure 3:**
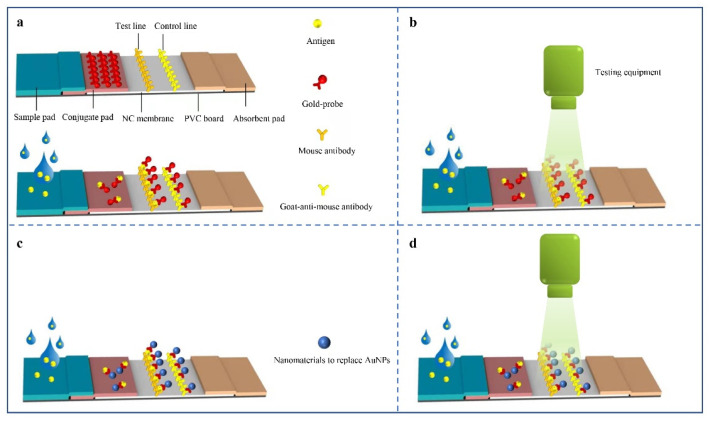
(**a**) Structure and principle of the traditional colloidal gold immunochromatographic strip. Its detection principle is as follows: (1) the markers combine with gold-probes on the conjugate pad, to form complexes after the user drops positive samples; (2) then complexes are captured by the antibodies at the test line and generate a red line; (3) the gold-probes without binding marker are captured by the goat-anti-mouse antibodies at the control line. If the control line is not red, the gold-probes may be invalid. (**b**) Demonstrated a method for achieving sensitivity enhancement through an external detection device. (**c**) Represents a class of methods using other nanomaterials (such as carbon nanoparticles, carbon nanotubes, and gold nanocages) instead of AuNPs, to improve detection sensitivity. (**d**) Shows a combination of the methods shown in (**b**,**c**).

**Figure 4:**
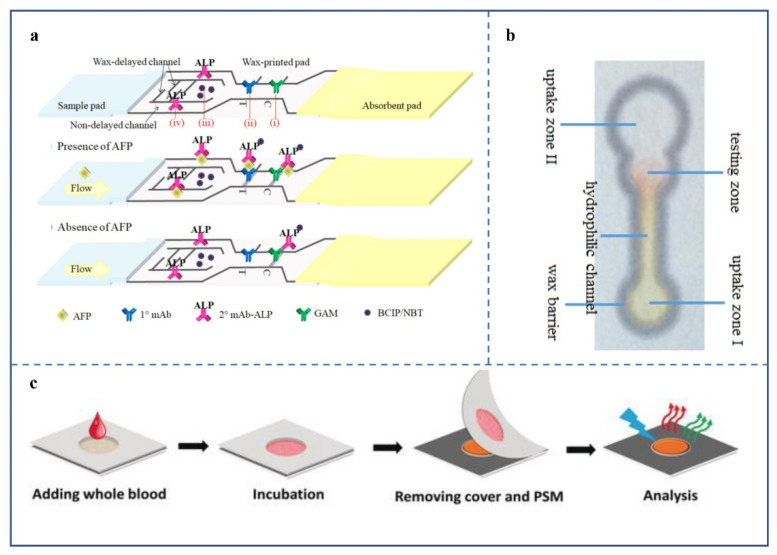
(**a**) Schematic diagram of μPAD structure and principle [56]. Before dropping the sample, ALP-labeled mouse antibody is in the no-barrier region, and BCIP/NBT is in the wax-formed barrier region. After dropping the positive sample, the ALP-labeled antibody reaches the detection line first, followed by BCIP/NBT, and chromogenic reactions occur. (Copyright © 2022 Published by Elsevier B.V.) (**b**) Channel structure of a μPAD for dopamine detection [57]. The structure includes a hydrophilic channel, uptake area, and test area around a hydrophobic wax barrier. The uptake area I and test area colors are reaction products (ferric chloride and dopamine) and phenanthroline. (Copyright © 2022 Elsevier B.V. All rights reserved). (**c**) Detection process of 4MC-loaded μPAD [58]. The device is simple to operate and has a wider range of applications. (Copyright © 2022–2022 John Wiley & Sons, Inc. All rights reserved).

Colorimetric analysis is one of the most commonly used methods in μPAD [52], and usually refers to the generation or change of color to present the detection results. The colorimetric method is widely used in μPADs because it is intuitive when reading. As early as 1961, colorimetry was applied to paper-based detection [53]. The most common paper-based detection products in life are colloidal gold immunochromatographic strips, with gold nanoparticles (AuNPs) and immune proteins binding as probes (Figure 3a). However, products are less sensitive [54]. As a result, some developers have begun to build matching reading devices, to improve sensitivity (Figure 3b). The data compiled by Calabria et al. showed that reading devices reduced the LOD of colloidal gold immunochromatographic strips from 50 ng/mL to 2 ng/mL [55]. Additionally, some researchers have replaced AuNPs with other nanomaterials (Figure 3c) or added detection devices after replacement (Figure 3d). Preechakasedkit et al. developed a μPAD with a LOD of 1 ng/mL of alpha-fetoprotein (AFP), by combining enzyme-linked immunosorbent assay (ELISA) with lateral flow immunoassay (LFIA) (Figure 4a) [56]. Liu et al. [57] reported a μPAD using an oxidation-reduction method. The device achieved a LOD of 0.37 μmol/L for dopamine with the help of Photoshop software (Figure 4b).

Although colorimetry has many advantages in the qualitative case, it is unsatisfactory in the quantitative case. In addition, some subtle color changes need to be observed by reading devices, which to some extent hinders the portability of μPADs. Thus, the simultaneous improvement of colorimetry-based μPAD sensitivity and portability may require the help of more accurate and compact color analysis equipment.

### 3.2. μPADs Based on Fluorescence

With the development of fluorescent nanoparticles [59,60,61,62,63,64], such as quantum dots, fluorescent-silica nanoparticles, and fluorescent-polystyrene microspheres, fluorescence has been widely used in the detection field [65,66]. Researchers have used physical or chemical methods to attach fluorescent nanoparticles to biomolecules that bind specifically to the target molecule. The inspector can detect the target molecule by observing the fluorescence emitted from the detection area. Luo et al. developed a paper-based ratio fluorescence analyzer for high-precision detection of human serum albumin (HSA) [58]. The device was loaded with 4MC (2′-hydroxychalcone derivative), and HSA-induced decomposition of 4MC nanoaggregates into 4MC-HSA complexes resulted in the fluorescence changing from red to green (Figure 4c). In devices based on LFIA, many strips use fluorescent particles to replace AuNPs in the colloidal gold immunochromatographic strips. They are equipped with corresponding detection equipment, which improves the detection sensitivity and allows quantitative analysis. In addition to its combination with immunoassays, fluorescence is also used as a primer marker for paper-based nucleic acid assays [67].

However, this method relies heavily on external devices (fluorescence excitation and detection devices), making the μPADs less portable. This disadvantage will make it hard to apply relevant products for home self-examination, resulting in a reduced market capacity. In addition, the production conditions of some fluorescent nanomaterials are very demanding. In the case of QDs, their synthesis requires high temperatures, and the absence of oxygen and water. Therefore, in addition to small and sensitive detection devices, researchers also need to develop stable and simple fluorescent nanomaterials.

### 3.3. μPADs Based on Chemiluminescence

**Figure 5:**
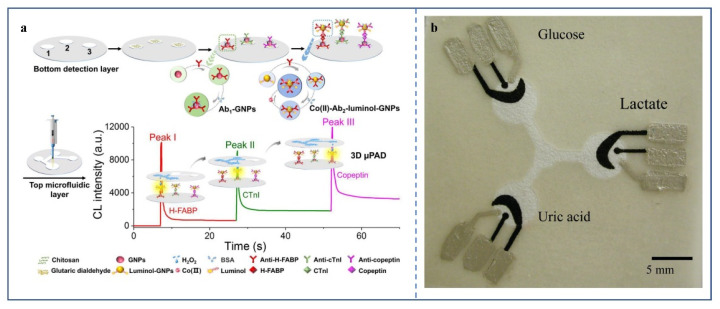
(**a**) Structure and the principle of 3D μPAD for H-FABP, cTnI, and copeptin multiplexed CL immunoassays [48] (Copyright © 2022 Elsevier B.V. All rights reserved). (**b**) Picture of first μPAD based on electrochemical signals [15]. The center of the white area is hydrophilic and draws the sample into three reaction zones containing electrodes. The silver electrode and contact pad are made of Ag/AgCl paste, and the black electrode part is a lead-modified carbon electrode. (Copyright © 2022, American Chemical Society).

As a detection and analysis method, chemiluminescence has a development history of many years. It works by making a chemical reaction in which electrons jump from their ground state to an excited state, and then return to the ground state to emit light [68]. Many chemiluminescence-based microfluidic detection devices have been reported [69,70]. The combination of chemiluminescence and low-cost μPADs has been reported [71,72]. Among them, the μPAD made by Li et al. can simultaneously detect three significant markers of acute myocardial infarction: heart-type fatty acid-binding protein (H-FABP), cardiac troponin I (cTnI), and copeptin [48] (Figure 5a). The sensitivity is excellent, and the LOD of the three markers are 0.06 pg/mL, 0.3 pg/mL, and 0.4 pg/mL, respectively.

Chemiluminescence analysis not only has strong anti-interference and quantitative analysis ability but also does not require an excitation light. These features are well suited for μPADs. While there are many advantages to a μPAD based on this method, few related products are available. This may be caused by the fact that they are not competitive with other techniques in price.

### 3.4. μPADs Based on Surface-Enhanced Raman Spectroscopy

Surface-enhanced Raman spectroscopy (SERS) is a technology that enhances the surface sensitivity of Raman scattering using molecules adsorbed on rough metal surfaces or nanostructures, such as plasma magnetic silica nanotubes [73]. This technology has a high sensitivity [74,75] and enables detection at a single-molecule level [76]. However, due to the complex composition of clinical samples, the enhanced surface often does not function as expected on paper materials. Therefore, the application of SERS in μPADs is limited to a certain extent. Siebe et al. developed a simple, novel spray-deposition technique, to manufacture SERS-active paper substrates [77]. The technology achieved a high purity of the sample and strength of surface enhancement on a single paper. This work extended the application of SERS in μPADs.

### 3.5. μPADs Based on Electrochemical Signal

Dungchai et al. produced the first μPAD based on electrochemical signals and using photolithography and screen printing [15] (Figure 5b). Electrochemistry has significantly improved the sensitivity of μPADs, and researchers in this field have published many results. [78,79]. The method can be divided into two categories: one is electrical signal detection, and the other is electrochemiluminescence.

An electrical signal is a voltage or current that changes over time. Some biochemical reactions at an electrode can cause a change in current. By detecting current changes, a biological analysis can be implemented. The detection method does not depend on color and avoids the interference of some colored substances in the sample. In recent years, the most representative result of this method in paper-based detection is a device for detecting SARS-CoV-2 antibodies [49]. The device can detect antibodies in human serum with high sensitivity. However, because of the need for external electrical signal detection equipment, there is still room to optimize its portability.

Electrochemiluminescence (ECL) is a high-efficiency analysis technology [80], which not only has no background light noise, as in chemiluminescence, but can also control the reaction using an electric current [81]. The selectivity of ECL analysis is improved by changing the electrode voltage to prevent oxidized/reduced substances from participating in electrochemiluminescence reactions. This usually uses ruthenium compounds, especially tris (bipyridine) ruthenium chloride (II) ([Ru (bpy) 3]^2+^) (releasing photons at ~620 nm) and reacts with tripropylamine to achieve regeneration. ECL has a more straightforward optical setting than photoluminescence. Compared with chemiluminescence, this process can realize reasonable control of time and space. The use of ECL makes μPADs excellent for metal ion identification [82] and biomarker detection [83].

## 4. Applications of μPADs in IVD

IVD refers to detecting certain samples (urine, blood, secretions, free cells, etc.) outside the human body, to obtain diagnostic information [84]. IVD-related technologies have developed rapidly, from the cellular level to the molecular level. The rise of POCT [85] in recent years, has brought IVD to a new level, making it time-effective and easy to operate [86].

The introduction of microfluidic technology has observably promoted the progress and market development of POCT. Especially paper-based microfluidic devices, effectively reducing the cost of POCT, making the detection process more straightforward [87,88] and the possible uses broader [89].

### 4.1. Main Applications of μPADs in Microbial Infection Diagnosis

Infectious diseases are a significant threat to human health in today’s world [90]. Most contagious diseases are local or systemic inflammation or organ dysfunction caused by pathogens, such as pathogenic bacteria and viruses, and having greater harmfulness and higher mortality. At present, the incidence rate of infectious diseases has increased, and pathogens are showing a trend of diversification and increased complexity [91,92]. Severe acute respiratory syndromes, such as COVID-19, and H7N9 avian influenza, are emerging. Some broad-spectrum drug-resistant pathogens have revived or developed new pathogenic features. For infectious diseases, timely detection is critical for disease treatment and epidemic prevention and control. However, some pathogens are mutating and spreading faster than they can be controlled. Therefore, the requirements for the accuracy and timeliness of the diagnosis of infectious diseases are higher in clinical practice, and the development of instant diagnosis caters to this demand [93].

Traditional methods for identifying pathogenic microorganisms, such as smear microscopy, in vitro culture, and mass spectrometry, have the disadvantages of a long cycle, high cost, complex operation, and limited detection location [94]. However, μPADs have excellent potential in microbial infection detection. They directly use human body fluids to complete real-time diagnosis, without a complex operation.

**Figure 6:**
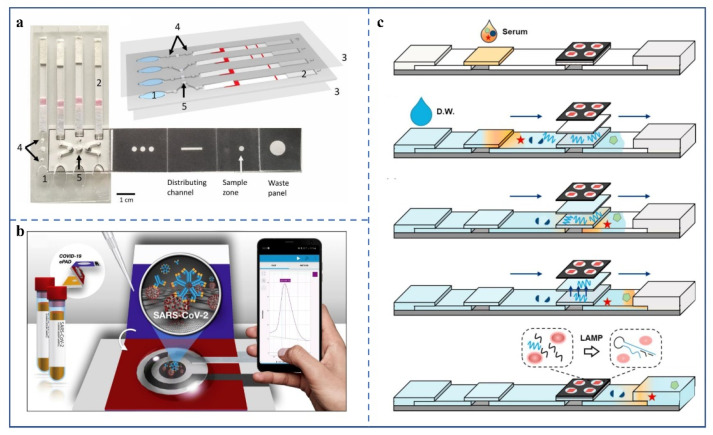
(**a**) A schematic diagram of an μPAD used for nucleic acid detection [99]. 1 is a buffer chamber: it generates power by pressing. 2 is a lateral flow test strip. 3 is an acetic acid film, its role is to seal the chip. 4 is the paper valve, it prevents the LAMP reaction system from interfering with other areas. 5 is the origami area for LAMP reaction. (Copyright © 2022 the Author(s). Published by PNAS) (**b**) There is a schematic illustration of the device components, detection principle, and detection procedure of the device [49]. (Copyright © 2022 Elsevier B.V. All rights reserved). (**c**) The μPAD operation principle and steps [101]. (1) The serum is mixed with dried lysis buffer after dropping the serum sample. The cleaved virus releases RNA molecules. (2) Distilled water (D.W.) is dropped for washing. RNA from the serum sample is transported to the amplification zone by the lateral flow. (3) The target RNA is purified and concentrated on a chitosan-dried binding pad at the bottom part of the amplification zone. (4) Target RNA is released by increasing the pH. During the lateral flow, Tris-HCl components induce a pH increase, to release the target RNA from chitosan. (5) RT-LAMP reactions for specific targets are conducted in each reaction pad. (Copyright © 2022 Elsevier B.V. All rights reserved).

The microfluidic devices used for microbial infection detection mainly adopt immunoassay and nucleic acid assay. After the pathogen infects the body, the body will generate corresponding antibodies, according to the antigen determinant of the pathogen. Therefore, the infection of the detected person can be indirectly known by detecting the specific antibodies in human blood [95,96]. Colloidal gold immunochromatographic strips used to detect IgG and IgM in human blood samples were reported soon after the outbreak [97]. However, unlike nucleic acid tests, antibody tests cannot be used in the early stages of infection, because the body does not produce enough antibodies for detection until 3–5 days after infection with the pathogen. μPADs for nucleic acid detection, combining isothermal nucleic acid amplification technology, have also been developed [98,99,100]. For example, Reboud et al. reported on a unique μPAD, used to detect viral DNA (Figure 6a) [99]. The device is divided into three functional areas: buffer room, foldable strip, and lateral flow strip. The buffer chamber provides power for the liquid to flow when pressed. The foldable strip is for DNA extraction and isothermal amplification, with holes of different shapes and functions in each layer. The lateral flow strips use LFIA to present detection results. This method, which combines isothermal nucleic acid amplification technology with LFIA, enables μPADs for nucleic acid detection that are simpler to operate, faster to detect, and can be used in a broader area. However, additional heating apparatuses are needed to reach temperatures suitable for isothermal nucleic acid amplification. The trend is to improve the independent operational capability of these μPADs. Designing self-heating materials (such as reduced iron powder) into the μPADs will help solve this problem.

#### 4.1.1. Viral Infection

In IVD, rapid detection of infectious viruses is an important task. Especially during a global pandemic, government officials and medical personnel are deeply aware of the importance of rapid detection. The μPADs provide an efficient and low-cost method for this goal.

##### SARS-CoV-2

The SARS-CoV-2 infection has caused harm to many people [102]. There are many traditional methods for nucleic acid tests [103,104], which are time-consuming and require expensive equipment. To overcome these shortcomings and improve diagnostic efficiency, the research and development of microfluidic devices for diagnosing SARS-CoV-2 infection has become one of the main tasks of certain research groups [105,106]. Among them, LFIA-based tests for SARS-CoV-2 are already on the market, but the sensitivity of this method is relatively low. Researchers are working on its sensitivity [107,108].

Kasetsirikul et al. reported two similar methods for detecting anti-SARS-CoV-2 antibodies using an enzyme-linked immunosorbent assay (ELISA) on paper [109,110]. The recombinant SARS-CoV-2 nucleocapsid antigen was coated on a paper device prepared by the lamination method. Then empty sites were closed with bovine serum albumin, and then the process of ELISA was performed, to detect the target antibody. It is more sensitive than commercial ELISA kits, thanks to image analysis devices. Therefore, the sensitivity and portability still need to be improved. In addition, using ELISA, a μPAD was developed by Gong et al. for quantitative antibody detection [111]. The tension rotating gyro platform collects the serum to make the device sensitive for the early diagnosis of disease. By detecting clinical samples from early-stage patients, this method has proven successful and has good application prospects.

To further improve the reliability of immunoassay, multiple antibodies against SARS-CoV should be detected simultaneously. With a sandwich immunoassay and antigen specificity testing of antibody cross-reaction, a paper-based sensor device that can distinguish different antigens, including the spinous process protein variant of SARS-CoV-2, has been developed [112]. The device can establish the binding mode, by changing the number, arrangement, and specificity of antibodies coated on paper, to distinguish the spinous process antigens of different coronaviruses.

Compared with the above methods, the labeling-free detection implemented on a paper-based microfluidic platform is more convenient. Yakoh et al. invented a paper-based electrochemical detection platform for SARS-CoV-2 antibodies (Figure 6b) [49]. Its main feature is that it does not need specific antibody labelling. It changes the current intensity after the SARS-CoV-2 antibody binds to the antigen in the positive sample and finally displays the test results through external equipment. The LODs of IgG and IgM were 0.96 ng/mL and 0.14 ng/mL, respectively. In clinical trials, the detection results of the platform were almost the same as those of ELISA kits, which met the clinical requirements. In addition, the team also extended the platform to detect spike proteins. The team has not yet rigorously verified the quantitative performance of the method, so it may not have an absolute competitive advantage in the market.

Interleukin-6 (IL-6) has become one of the biomarkers used to detect the severity of COVID-19 [113]. Adrover-Jaume et al. concluded from published research results that the serum IL-6 level in mild cases was between 5.1 and 18.8 pg/mL [114]. While in moderate or severe cases, the value increased to 22.5–198 pg/mL. Based on this, his team developed a μPAD to detect IL-6 in the serum of patients infected with SARS-CoV-2. The device combines a paper-based signal amplification mechanism with a program for color quantization. Through this design, when the sample contains an ultra-low concentration of IL-6, the machine can also produce a stable colorimetric signal. The LOD of this method is 10^−3^ pg/mL, which is a superior qualitative performance. However, its quantitative ability needs to be improved for more accurate detection.

Due to the limitations of traditional nucleic acid detection methods, some researchers have also begun to strive to realize the efficient detection of nucleic acid on a μPAD. A device with excellent sensitivity and specific nucleic acid detection and based on paper has been developed [115], which can detect SARS-CoV-2 through human saliva within 60 min. The loop-mediated isothermal amplification (LAMP) [116], which is more advantageous than PCR for rapid detection, is used in the device.

By applying aptamer to LFIA, researchers have completed nucleic acid assays. However, the extraction or amplification of nucleic acid needs to be carried out by traditional means, which does not completely overcome the shortcomings of conventional methods. Yu et al. used this approach to develop a nucleic acid detection device [117]. The nucleotide sequence obtained by single-tube reverse transcription-polymerase chain reaction (RT-PCR) was detected on a lateral flow test strip. The device can detect RdRp, ORF3a, and N genes simultaneously. The LOD of each gene is ten copies/time. Previously, a SARS-CoV-2 detection method using RT-PCR and then paper chromatography was reported [67]. The technique uses cas12 to cut a single-stranded DNA probe with biotin (Bio) and fluorescent groups modified at both ends, then the test strip is inserted into the solution, and finally the detection result is judged by observing the presence or absence of fluorescent stripes on the test line.

##### Zika Virus

Zika virus (ZIKV) has been identified as the cause of severe neurological complications in humans [118]. About one in five infected people will develop symptoms after an incubation period of about 3 to 12 days, after being bitten by mosquitoes carrying ZIKV. Six years ago, a ZIKV outbreak highlighted the need for quick, low-cost tests; and paper-based tests are one way to meet these requirements [119].

Pardee et al. reported a paper sensor device for detecting the ZIKV RNA genome [120]. The device combines nucleic acid amplification and detection. Nucleic acid sequence-based amplification [121] was used to amplify virus RNA in this device. When the RNA content reaches the LOD, the color in the detection area changes from yellow to purple. The device can reduce the cost of detection to a certain extent, but it still cannot achieve fast and convenient detection.

To quickly and easily detect ZIKV, Karaj et al. developed a paper-based microfluidic chip using wax printing technology [122]. The chip completes nucleic acid amplification by reverse transcription-loop-mediated isothermal amplification, a more direct thermostatic amplification technology. In addition, the group studied and optimized the paper types, pore sizes, and channel sizes, to ensure that untreated biological samples (undiluted human urine and diluted human plasma) are filtered properly during the capillary action-driven flow. The chip is easy to operate and can detect samples with virus concentrations greater than or equal to 1 copy/μL. However, the hot plate used in this method needs to be cleaned before each test, to prevent cross-contamination, which is hard to apply in continuous testing. Therefore, some anti-contamination components need to be added to the chips themselves.

Draz et al. developed a μPAD based on paper, using electrical sensing and nanoparticle signal amplification technology [123]. The device obtains microelectrodes through screen printing. First, the viruses are isolated by immunomagnetic beads and then bind to the platinum nanoparticles (PtNPs) to amplify the detection signal. After the captured ZIKV-PtNP complex is dissolved with detergent, the released charged molecules and PtNP change the conductivity of the solution. The group obtained the test result by observing the change in current. However, the detection process of the device is complicated. Thus, researchers should improve the automation of the device.

A more integrated μPAD (Figure 6c) has been demonstrated [101], requiring only 10 μL–50 μL human serum, that can make an all-in-one molecular diagnosis of ZIKV, dengue virus, and Chikungunya virus. The whole process of virus detection, including sampling, extraction, amplification, and detection, can be performed on a paper device, which is very simple and efficient. However, the detection time of the device is too long, at about 60 min. Thus, reducing the detection time to less than 30 min would benefit its market competitiveness.

##### Hepatitis B Virus

Hepatitis B virus (HBV) is not only the cause of hepatitis B, but also a leading cause of chronic liver cirrhosis and hepatocellular carcinoma (HCC) [124]. The blood of infected people is infectious, whether in the latent, acute, or chronic phases. Most HBV detection methods require large professional instruments. Miniaturization of detection equipment has been carried out continuously over the past 20 years, and paper-based microfluidic devices are one of its directions.

Based on the significant advantages of ECL in rapid detection, Chen et al. developed a new HBsAg detection platform with great potential [125]. Compared with chemiluminescence immunoassays [126] and ELISA, this platform showed a more satisfactory sensitivity and efficiency. Its disadvantage is that the acquisition of detection results requires a larger device. Using mobile devices such as smartphones to obtain test results will reduce the cost of the device and improve portability, thus expanding its market prospects.

In an immune paper chromatography study [127], a group used highly luminescent quantum dot beads as tracer signals, which are more sensitive than traditional colloidal gold immunochromatography. The device is ultra-sensitive (the LOD is 75 pg/mL) and can achieve quantitative detection of the hepatitis B virus surface antigen (HBsAg) in human serum. However, the combination of labeled antibodies and antigens requires additional operations before the device runs. Thus, a functional area responsible for this process should be added, to improve device automation.

In 2016, Sanjay et al. developed a hybrid microfluidic chip based on paper and polymethyl methacrylate [128]. The device enables low-cost and high throughput diagnosis of hepatitis B. IgG and HBsAg were detected by alkaline phosphatase combined with secondary antibody and BCIP/NBT. One hour after the sample is added, the user can obtain the test result with the naked eye. The LOD of IgG and HBsAg is 1.6 ng/mL and 1.3 ng/mL, respectively. The chip has a lot of room for improvement, both in detection speed and sensitivity.

Aydin et al. developed a type of paper chip for detecting HBV DNA, using conjugated polyelectrolytes that change color with the combination of primers and target DNA [129]. Through the conformation transformation of the nucleic acid-CPE complex, the paper chip produces an obvious colorimetric response, so that the human eye can intuitively obtain the detection results. For quantitative analysis, the team also proposed a method of pixelating color, which can accurately quantify the concentration of nucleic acid detection.

Another team [130] proposed a μPAD that can perform a multi-step operation in a single device for the first time. It has good selectivity and is for unlabeled HBV DNA detection. The working electrode of the μPAD captures target DNA using covalently immobilized pyrrolidine nucleic acid with high affinity and selectivity. [131]. DNA causes the electrochemical signal of ferrocyanide (III)/(II) to vary with concentration. The LOD of the device is 1.45 pm, which provides a referential method for DNA detection.

##### HIV

HIV is the pathogen of AIDS and spreads through body fluids. Among the HIV testing products on the market, the LFIA-based products have the highest popularity, because of their convenient use and low cost. In addition, researchers have developed some μ pads for HIV testing that can also meet POCT requirements.

In POCT, LFIA is widely used for its speed and simplicity, but it has the disadvantage of low sensitivity, which hinders its further popularization. Many researchers are committed to solving this problem. Tang et al. integrated a concentration method into LFIA. They used the technology to detect HIV nucleic acid with good results [132]. The results showed that a 10× signal enhancement was completed in less than 25 min. Dector et al. first proposed a paper-based microfluidic cell fueled by blood [133]. They integrated it into LFIA as the power source for independent HIV detection. The anode is glucose oxidase, and the cathode is a platinum electrode. The top is provided with two windows for storing samples and contacting air.

Lu et al. constructed a flexible paper-based electrode based on a membrane, to detect HIV by DNA hybridization [134]. The membrane electrode material is Ni-Au complex/carbon nanotube/polyvinyl alcohol. It realized the efficient combination of a metal-organic skeleton and single-stranded DNA and improved the detection sensitivity. However, the complex production process may hinder its industrialization.

**Figure 7:**
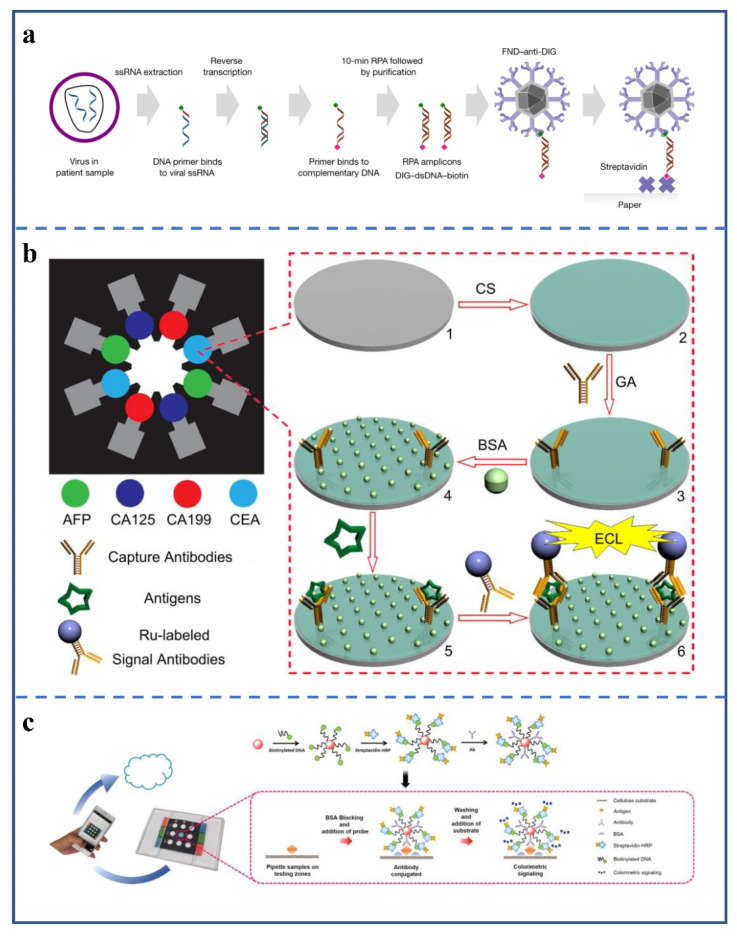
(**a**) Schematic of the detection principle [135]. Viral RNA is amplified using primers modified with DIG and biotin, respectively. One end of the amplicon is bound to the FND surface antibody and the other end to streptavidin on the test strip, forming a sandwich structure when positive samples are detected. (Copyright © 2022, The Author(s), under exclusive license to Springer Nature Limited). (**b**) Schematic representation of the fabrication and assay procedure for a 3D paper-based ECL device [50]. (1) screen-printed carbon working electrode; (2) after chitosan modification; (3) after immobilization of capture antibodies; (4) after blocking and washing; (5) after capturing and washing; (6) after incubation with signal antibodies, washing, and triggering ECL reaction. (Copyright © 2022 Elsevier Ltd. All rights reserved). (**c**) Schematic diagram of paper-based detection using a functionalized gold nanoprobe sensing strategy [137]. The preparation principle of the probe and the detection process of the method. The results can be identified and uploaded via a smartphone. (Copyright © 2022, American Chemical Society).

Miller et al. reported a μPAD for ultrasensitive detection of HIV. The device uses fluorescent nanodiamond particles (FNDs) as an indicator label (Figure 7a) [135]. When in use, first, the single-stranded RNA in HIV is extracted, and the primer with digoxin (DIG) at the 5′ end is used for reverse transcription. The reverse-transcribed DNA is copied with the primer with Bio at the 5′ end to produce double-stranded DNA with DIG and Bio at both ends. The samples, after two transcription operations, are subjected to immunochromatography. If samples are positive, the double-stranded DNA will first bind to the FNDs linked with the anti-DIG antibody and then attach to streptavidin (SA) on the detection line. One SA has four Bio-binding sites, which finally makes more FNDs stay at the detection line, further improving the detection sensitivity. The nitrogen-vacancy center-defects of diamond particles have good optical properties [136], when produced by high-energy particle irradiation.

#### 4.1.2. Bacterial Infection

Pathogenic bacteria infect the human body through food, air, sexual contact, and in other manners. In less developed areas, bacterial infection has become one of the main threats to human life. In the past decade, it has caused the highest mortality in developing countries [138], especially among children [139]. In addition to directly threatening human life, the risk of bacterial infection to human health is often potentially chronic. For example, Helicobacter pylori will increase the incidence of gastric cancer in infected people after causing ulcers [140]. Therefore, the realization of convenient and rapid bacterial detection, would not only help to find and eliminate pathogens quickly, but would also be helpful for the early prevention of some diseases. μPADs have great potential in detecting bacterial infection, because of their fast detection speed and easy use.

A research team [141] used 5,5-dithio-bis-(2-nitrobenzoic acid) combined with AuNPs to generate a strong Raman signal. Testing personnel completed the SERS test using a lateral flow chromatography test strip, which can realize the effective detection of target bacteria. In a comparative study of the detection performance of buffer and serum samples, the method maintained good sensitivity for serum detection. However, the standard detection time data for serums with different concentrations were not discussed in detail by the authors.

He et al. developed an μPAD for early screening of bacterial infection [142]. The functional pattern of the device is prepared using a laser, and the detection result is determined by observing the change of color with the naked eye. The chromogenic agar in a lower layer of the device identifies the desired pathogens; the cellulose filter paper in the middle layer distributes the sample evenly; the cellulose filter paper hole on the top layer contains different doses of antibiotics for drug sensitivity testing. Experiments with *E. coli* showed that the timeliness and effectiveness of the device met the requirements of POCT.

Kim et al. demonstrated a μPAD for detecting highly pathogenic *E. coli* in feces, which functioned by double staining and analyzed using RGB [143]. Its sensitivity meets practical needs. Moreover, due to its low cost, it will be welcomed by less developed countries and regions. However, the device is not sufficiently portable. As a result, its market space may be squeezed by more portable and simpler products.

A research group that developed a paper-based ELISA for rapid identification of *E. coli* recently reported a turntable paper-based detection equipment with more straightforward operation [144]. The device is composed of an acrylic base at the bottom, a wooden chopstick rotating shaft in the middle, and three layers of paper. The second layer of paper (fixed on the rotating shaft and can be rotated) is printed with wax to form a circular reaction zone. The third layer (nonrotatable) is cut into a circular plate shape and divided into six hydrophobic test areas by resin. The sample to be tested is loaded into the reaction area of the second layer of paper, and the reagent required for paper-based ELISA is gradually added to the hydrophobic boundary test area on the top layer of the third layer. After repeated rotation and cleaning, the image is detected using a smartphone. Although the paper-based system is convenient for obtaining results, the complex operation process needs to be simplified. In addition, the research group did not prove the application potential of the device through clinical experiments, so its usefulness needs to be evaluated. Before this work, Li et al. reported paper-based ELISA colorimetry to diagnose brucellosis [145]. The method requires 5 mL of serum and the detection time is 2 h. In a real POCT scenario, these data are not very satisfactory, but the clinical detection ability of this method was verified in the detection of serum samples.

Applying more sensitive electrical signal analysis to PAD, Alatraktchi et al. produced a working electrode, counter electrode, and reference electrode by screen printing [146]. They covered the electrode with iron/ferrocyanide as the redox probe. The electrodes were connected to a portable potentiostat via a three-pin connector. The team used the device to detect pseudomonas aeruginosa (PA). The marker detected by the device is pyocyanin (PYO), which is a specific marker highly related to PA. Another group [147] reported a paper-based piece of analysis equipment that can detect PA with high sensitivity using saliva, and which also catches PYO by electrochemical means, to obtain infection information. Its LOD is as low as 10 nmol/L, but the study used substitutes for clinical samples. Therefore, the device needs to participate in multiple clinical trials to verify its performance.

### 4.2. Main Applications of Microfluidic Devices in Tumour Detection

Tumor diseases seriously threaten human life safety, especially malignant tumors, with a low cure rate and high treatment cost. As for as the existing medical technology, early screening and diagnosis are the most effective treatments for tumor diseases. Detecting tumor markers is the most promising way to realize early tumor screening. At present, imaging screening is still the primary method of tumor detection. However, imaging screening has a lag and can fail to diagnose early cancer, making patients miss the best time for treatment. Therefore, many researchers began to focus on detecting tumor markers, to compensate for the possible misdiagnosis of imaging screening. With the deepening of relevant research, various tumor markers have been separated and applied [148,149,150,151]. The existing mainstream detection methods of tumor markers are time-consuming and costly. To achieve the popularization of early cancer screening, accurate, sensitive, and low-cost tumor marker detection methods [152,153,154,155,156,157] are essential. Paper-based detection methods to accomplish these purposes have been reported [158,159,160,161,162].

Liu et al. proposed a novel washing method that can effectively remove nonspecific proteins from μPADs and apply them for paper-based colorimetric analysis [46]. The experimental results showed that the method significantly increased and reduced the background interference, and the LOD of carcinoembryonic antigen (CEA) in serum was 0.03 ng/mL. However, the team used too few serum samples and did not test enough clinical samples. Therefore, further experiments are needed to prove its practicality. Mahmoudi et al. also developed a paper-based method for CEA detection, which only took 15 min to complete [163]. Detection performance was verified by sufficient clinical samples, showing great application potential.

Preechakasedkit et al. developed a μPAD for ELISA. Compared with traditional ELISA, the chip only needed to drop the sample, without other operations [56]. In this chip, alkaline phosphatase (ALP)-labeled AFP antibody and BCIP/NBT are in two channels. The former channel is unblocked, and the latter has carved channels formed by wax materials. As a result, the ALP-labeled antibody flows faster than BCIP/NBT after dropping the serum sample. The immune complex formed with AFP first reaches the test line, then BCIP/NBT arrives to complete the reaction. Experimental results showed that the limit of AFP detection in serum was 1 ng/mL, but these data do not have an absolute advantage in paper-based microfluidic chips. The limited sensitivity may also be related to the loss of some samples in the BCIP/NBT channel.

A μPAD with immune sensing technology was developed to overcome the difficult-to-spot medical pain stage in the early stages of pancreatic cancer [164]. The marker is the pseudopodium-enriched atypical kinase 1 (PEAK1). Graphene oxide was immobilized on an electrode and then labelled with an anti-PEAK1 antibody, to construct the μPAD. When the device detects the target substance, a double antibody sandwich structure is formed, and detection is completed using an electrical signal. Since the existing methods for early diagnosis of pancreatic cancer are unreliable, the emergence of such technology is of practical significance for promoting the progress of early screening of this type of cancer. Another paper-based method for sensitive detection of PEAK1 was created [165] that directly observed the results by colorimetry. This technique, utilizing the chemical characteristics of AuNPs that can catalyze the degradation of color dyes, can complete the detection process without specific instruments. However, a single marker does not have an absolute reference value for cancer diagnosis, so the researchers should upgrade the single-indicator detection of the equipment to multi-indicator detection.

Jiao et al. invented a 3D vertical flow μPAD, using the double antibody sandwich method [47]. Its 3D structure is formed by paper folding, divided into a sample layer, cleavage layer coated with fluorescein isothiocyanate labelled antibody, and detection layer coated with monoclonal antibodies specifically binding to the marker to be tested. Each layer has different shaped functional areas. The device can realize the simultaneous detection of various cancer markers. The results showed that the device has a high sensitivity and selectivity for detecting CEA, AFP, and cancer antigen 199 (CA199), with LODs of 0.03 ng/mL, 0.05 ng/mL, and 0.09 U/mL in human serum, respectively. However, the concentrations of the three markers in the blood of healthy people were higher than the device’s detection limit. Therefore, the device needs to improve its quantitative ability or generalize the image features of the detection results corresponding to each marker when it exceeds the standard value.

Ge et al. used Ru-labeled antibodies as signaling antibodies to complete electrochemical signal detection on paper (Figure 7b) [50]. Its structure is a star strip, the middle of which is the sampling area, and the end of each branch is not detected. They detected CEA, AFP, CA125, and CA199, using four color light signals, respectively. The LODs of the four markers were 0.5 ng/mL, 0.15 ng/mL, 0.6 U/mL, and 0.17 U/mL, respectively. However, the different detection areas may interfere with each other, limiting its sensitivity. The sensitivity could be improved if the μPAD could achieve different time periods for each marker or signaling antibody in reaching the detection area.

Chu et al. provided a paper-based chemiluminescence technique for multiple detection of tumor markers [166]. In the experiment they reported, three bifurcation and detection regions were designed for CEA, (carcinoma antigen 125) CA125, and CA199, respectively, with good repeatability. Chitosan coating and glutaraldehyde crosslinking methods were used to fix the capturing antibodies of each marker in the corresponding detection region. Each fork has a barrier made of sucrose, and the number of barriers varies; except that one fork has no barrier. The method prevents each detection area from generating signals simultaneously, avoiding interference between them. The LODs of CEA, CA125, and CA199 in serum were 0.03 ng/mL, 0.2 U/mL, and 0.2 U/mL, respectively. The technology has the potential to be made into a commodity in the market, but researchers should increase the number of detection channels, to detect more markers at one time.

Wang et al. reported a μPAD using a nucleic acid aptamer and electrochemical detection, with high sensitivity and specificity [167]. The μPAD is made using batik and screen-printing technology, to realize the functions of sample filtering and automatic sample injection. There are two detection areas in the lower layer of the device, which are used to detect CEA and neuron-specific enolase, respectively. The detection results are characterized by differential pulse voltammetry. Although the clinical testing of samples has yielded excellent results, it remains unproven whether it can be used for the early detection of cancer. Therefore, large-scale testing of clinical samples and the follow-up of suspected early patients are needed.

In another study based on electrical signals to detect cancer-related nucleic acids [168], Signal ON and Signal OFF were used in a paper-based microfluidic chip for detecting breast cancer-specific nucleic acid sequences, respectively, and compared. The chip is connected to a three-electrode system. The reference electrode is made of Ag/AgCl ink, and the working electrode and counter electrode are made of graphite ink. Colloidal gold solution is dispersed on the working electrode, so that the DNA probe is fixed on the paper chip through a thiol-gold bond. The results showed that the two methods had little difference in detection performance. However, in practice, both have their advantages and disadvantages. For quick field applications that require POCT, signal OFF represents the best compromise between manufacturing complexity and ease of use.

A μPAD for detecting cancer markers in 15 min was developed, to speed up early cancer screening [137]. The probe was made by modifying AuNPs three times (Figure 7c). In the first modification, AuNPs were modified with nucleotides containing continuous poly adenine sequences connected with Bio. Compared with the Au-S bond, this technology has a more vital binding force, faster preparation, and lower cost. The second modification used SA-HRP to bind nucleic acid via Bio. The third modification used antibodies to modify the surface of AuNPs for specific binding to antigen. After dropping the sample, the modified AuNPs were added, and finally, 3,3′,5,5′-tetramethylbenzidine was added. The diagnostic results were determined by color. They added different concentrations of kallikrein-3 to the serum for testing and obtained a LOD of 10 pg/mL. However, the work lacked data from actual clinical samples. In addition, the preparation of the probe in this method is complicated. Without simplification, the device would be hard to popularize.

## 5. Conclusions and Prospect

**Figure 8:**
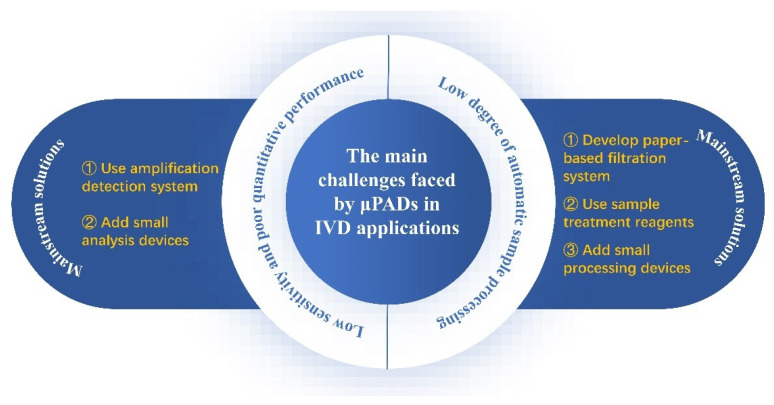
Main challenges and mainstream solutions for paper-based devices used in IVD.

In the future, the main development direction of μPADs is to develop cheap and easy-to-operate portable analysis devices, to expand the scope of application, especially in less developed areas. Therefore, IVD is developing towards POCT, characterized by miniaturization of instruments, simplification of operation, low detection price, and real-time reporting of results. The emergence of paper-based microfluidic technology has promoted the development of POCT in multiple dimensions, and part of the technology has also been transformed into commodities. However, μPADs still face some challenges in the application of IVD, which limit their promotion (Figure 8). Next, we will discuss some common problems in paper machine microfluidic devices.

For the diagnosis of disease, the sensitivity and specificity of the test tool are critical. These two parameters are closely related to the accurate and early diagnosis of disease, wherein developments in sample filtration, and immunological protein and aptamer preparation can increase the specificity of detection. However, μPADs have not become the mainstream clinical diagnosis, due to their limited sensitivity. There are two main reasons for their low sensitivity: (1) the effective concentration of the sample in the paper material is reduced; (2) the performance of available detection methods is limited. Most of the relevant research focused on the direction of sensitivity improvement. There are two basic approaches in which research results are abundant. One approach is to make electrical signal detection devices, fluorescence signal detection devices, and other small devices, to improve the signal acquisition ability. The most representative of this approach is the modification of electrodes on paper, which enables the paper devices to detect the target substance by changing the current generated by biological or chemical reactions. However, commercially available μPADs that detect electrical signals are rare, because much of the research remains proof-of-concept. In addition, the proliferation of expensive micro-detection devices may reduce the cost performance of μPADs. Therefore, the research and development of low-cost and high-performance micro sensors is of positive significance for the development of μPADs. Another approach is the chemical modification of paper materials, immune proteins, and tracers, which can improve the signal amplification independently of external devices. The most representative of this direction is the Bio-SA amplification system, which can improve detection sensitivity using the signal amplification advantages of one SA connecting four Bios with high affinity. The system has been used in mature applications, but a more stable and efficient amplification system needs to be developed to further improve the detection sensitivity of μPADs. Many researchers have combined these two directions and achieved a very high sensitivity. To be more suitable for clinical diagnosis, the high throughput of μPADs is also critical. Compared with 2D devices, 3D devices have a higher detection throughput. However, for quantitative detection, μPADs are still not accurate enough and require additional analytical equipment. In clinical practice, quantification of marker concentration is critical for disease diagnosis. Therefore, the field needs more reliable μPADs that can achieve a semi-quantitative or quantitative analysis of multiple biomarkers.

In addition to the above problems, the scope for automation applications of μPADs needs to be further explored. Although a paper material can realize sample flow through capillary force, its automation still needs external equipment in the case of sample processing. For example, for detecting viral nucleic acid, the virus in the sample needs to be broken up, and then the target nucleic acid is amplified and then tested on a paper chip or device. Improving the integration degree of the μPAD, such as adding a sample processing module, can simplify the operational steps and improve the integrated capability of the devices. This will further develop paper-based detection in the direction of automation. It is important to note that the additional functional components on μPADs need to be miniaturized, to ensure convenience and performance.

To sum up, μPADs overcome some challenges of IVD, promote the rapid development of this field, and make it develop towards POCT, which can better meet current demands. However, there is a lot of room for improvement. With the continuous emergence of new materials, methods, and processing technologies, the shortcomings of paper-based microfluidic technology will be overcome, and its advantages will be more prominent. In the future, μPADs will play a critical role in IVD and have great social benefits.

## Figures and Tables

**Table 1:** This table summarizes the processing methods for μPADs and their main advantages and disadvantages.

Production Method	Materials	Advantages	Disadvantages
Photolithography	SU-8 photoresist	high-resolution	high cost, complex operation, easy deformation
Wax printing	wax	low cost, simple operation	low resolution
Inkjet printing	alkyl ketene dimer	fast speed, good uniformity	requiring relatively large external equipment
Plasma etching	polymer layer	fast speed, small reagent consumption	high cost
Laser cutting	paper material	high precision, high efficiency	limited cutting thickness
Knife cutting	paper material	low cost, simple operation	low precision, poor uniformity
Flexographic printing	paper material	good flexibility, environmentally friendly	high cost, limited applications
Wax screen printing	wax	low cost, easy to operate	high occurrence of defects

**Table 2:** This chart shows the comparison of various methods used by μPADs.

Methods	Samples	Biomarkers	Capturing Principle	Limit of Detection	Test Time	Reference
Colorimetric analysis	Human serum	CEA	immunology	0.03 ng/mL	7 min	[46]
Fluorescence	Human serum	CEA	immunology	0.03 ng/mL	5 min	[47]
		AFP		0.05 ng/mL		
		CA199		0.09 U/mL		
Chemiluminescence	Human serum	H-FABP	immunology	0.06 pg/mL	30 min	[48]
		cTnI		0.30 pg/mL		
		copeptin		0.40 pg/mL		
Electrical signal	Human serum	SARS-CoV-2 IgG	immunology	0.96 ng/mL	30 min	[49]
		SARS-CoV-2 IgM		0.14 ng/mL		
Electrochemiluminescence	Human serum	CEA	immunology	0.5 ng/mL	2 h	[50]
		AFP		0.15 ng/mL		
		CA125		0.6 U/mL		
		CA199		0.17 U/mL		
Surface-enhanced Raman spectroscopy	Human serum	IL-10	immunology	0.1 pg/mL	4 h	[51]
		MCP-1		0.1 pg/mL		

## Data Availability

Data sharing not applicable. No new data were created or analyzed in this study.

## References

[1] Ma Q., Ma H., Xu F., Wang X., Sun W. (2021). Microfluidics in Cardiovascular Disease Research: State of the Art and Future Outlook. Microsyst. Nanoeng..

[2] Lee W.-C., Ng H.-Y., Hou C.-Y., Lee C.-T., Fu L.-M. (2021). Recent Advances in Lab-on-Paper Diagnostic Devices Using Blood Samples. Lab Chip.

[3] Iyer V., Yang Z., Ko J., Weissleder R., Issadore D. (2022). Advancing Microfluidic Diagnostic Chips into Clinical Use: A Review of Current Challenges and Opportunities. Lab Chip.

[4] Ayuso J.M., Virumbrales-Muñoz M., Lang J.M., Beebe D.J. (2022). A Role for Microfluidic Systems in Precision Medicine. Nat. Commun..

[5] He S., Joseph N., Feng S., Jellicoe M., Raston C.L. (2020). Application of Microfluidic Technology in Food Processing. Food Funct..

[6] Dhar B.C., Lee N.Y. (2018). Lab-on-a-Chip Technology for Environmental Monitoring of Microorganisms. Biochip J..

[7] Gao B., Yang Y., Liao J., He B., Liu H. (2019). Bioinspired Multistructured Paper Microfluidics for POCT. Lab Chip.

[8] Ming T., Luo J., Liu J., Sun S., Xing Y., Wang H., Xiao G., Deng Y., Cheng Y., Yang Z. (2020). Paper-Based Microfluidic Aptasensors. Biosens. Bioelectron..

[9] Noviana E., Ozer T., Carrell C.S., Link J.S., McMahon C., Jang I., Henry C.S. (2021). Microfluidic Paper-Based Analytical Devices: From Design to Applications. Chem. Rev..

[10] Martinez A.W., Phillips S.T., Butte M.J., Whitesides G.M. (2007). Patterned Paper as a Platform for Inexpensive, Low-Volume, Portable Bioassays. Angew. Chem. Int. Ed..

[11] Martinez A.W., Phillips S.T., Carrilho E., Thomas S.W., Sindi H., Whitesides G.M. (2008). Simple Telemedicine for Developing Regions: Camera Phones and Paper-Based Microfluidic Devices for Real-Time, off-Site Diagnosis. Anal. Chem..

[12] Martinez A.W., Phillips S.T., Whitesides G.M. (2008). Three-Dimensional Microfluidic Devices Fabricated in Layered Paper and Tape. Proc. Natl. Acad. Sci. USA.

[13] Carrilho E., Martinez A.W., Whitesides G.M. (2009). Understanding Wax Printing: A Simple Micropatterning Process for Paper-Based Microfluidics. Anal. Chem..

[14] Lu R., Shi W., Jiang L., Qin J., Lin B. (2009). Rapid Prototyping of Paper-Based Microfluidics with Wax for Low-Cost, Portable Bioassay. Electrophoresis.

[15] Dungchai W., Chailapakul O., Henry C.S. (2009). Electrochemical Detection for Paper-Based Microfluidics. Anal. Chem..

[16] Liu H., Crooks R.M. (2011). Three-Dimensional Paper Microfluidic Devices Assembled Using the Principles of Origami. J. Am. Chem. Soc..

[17] Liu L., Yang D., Liu G. (2019). Signal Amplification Strategies for Paper-Based Analytical Devices. Biosens. Bioelectron..

[18] Nguyen V.T., Song S., Park S., Joo C. (2020). Recent Advances in High-Sensitivity Detection Methods for Paper-Based Lateral-Flow Assay. Biosens. Bioelectron..

[19] Sarwar M., Leichner J., Naja G.M., Li C.Z. (2019). Smart-Phone, Paper-Based Fluorescent Sensor for Ultra-Low Inorganic Phosphate Detection in Environmental Samples. Microsyst. Nanoeng..

[20] Noviana E., McCord C.P., Clark K.M., Jang I., Henry C.S. (2020). Electrochemical Paper-Based Devices: Sensing Approaches and Progress toward Practical Applications. Lab Chip.

[21] Singh A.T., Lantigua D., Meka A., Taing S., Pandher M., Camci-Unal G. (2018). Paper-Based Sensors: Emerging Themes and Applications. Sensors.

[22] Lo S.-J., Yang S.-C., Yao D.-J., Chen J.-H., Tu W.-C., Cheng C.-M. (2013). Molecular-Level Dengue Fever Diagnostic Devices Made out of Paper. Lab Chip.

[23] Yamada K., Shibata H., Suzuki K., Citterio D. (2017). Toward Practical Application of Paper-Based Microfluidics for Medical Diagnostics: State-of-the-Art and Challenges. Lab Chip.

[24] Nishat S., Jafry A.T., Martinez A.W., Awan F.R. (2021). Paper-Based Microfluidics: Simplified Fabrication and Assay Methods. Sens. Actuators B Chem..

[25] Tong X., Ga L., Zhao R., Ai J. (2021). Research Progress on the Applications of Paper Chips. RSC Adv..

[26] Ozer T., McMahon C., Henry C.S. (2020). Advances in Paper-Based Analytical Devices. Annu. Rev. Anal. Chem..

[27] Han K.N., Choi J.S., Kwon J. (2017). Gold Nanozyme-Based Paper Chip for Colorimetric Detection of Mercury Ions. Sci. Rep..

[28] Tian T., Wei X., Jia S., Zhang R., Li J., Zhu Z., Zhang H., Ma Y., Lin Z., Yang C.J. (2016). Integration of Target Responsive Hydrogel with Cascaded Enzymatic Reactions and Microfluidic Paper-Based Analytic Devices (ΜPADs) for Point-of-Care Testing (POCT). Biosens. Bioelectron..

[29] Hu J., Xiao K., Jin B., Zheng X., Ji F., Bai D. (2019). Paper-Based Point-of-Care Test with Xeno Nucleic Acid Probes. Biotechnol. Bioeng..

[30] Qin X., Liu J., Zhang Z., Li J., Yuan L., Zhang Z., Chen L. (2021). Microfluidic Paper-Based Chips in Rapid Detection: Current Status, Challenges, and Perspectives. TrAC Trends Anal. Chem..

[31] Zheng W., Wang K., Xu H., Zheng C., Cao B., Qin Q., Jin Q., Cui D. (2021). Strategies for the Detection of Target Analytes Using Microfluidic Paper-Based Analytical Devices. Anal. Bioanal. Chem..

[32] Ge L., Wang S., Song X., Ge S., Yu J. (2012). 3D Origami-Based Multifunction-Integrated Immunodevice: Low-Cost and Multiplexed Sandwich Chemiluminescence Immunoassay on Microfluidic Paper-Based Analytical Device. Lab Chip.

[33] Martinez A.W. (2008). FLASH: A Rapid Method for Prototyping Paper-Based Microfluidic Devices. Lab Chip.

[34] Abe K., Suzuki K., Citterio D. (2008). Inkjet-Printed Microfluidic Multianalyte Chemical Sensing Paper. Anal. Chem..

[35] Li X., Tian J., Nguyen T., Shen W. (2008). Paper-Based Microfluidic Devices by Plasma Treatment. Anal. Chem..

[36] Fenton E.M., Mascarenas M.R., López G.P., Sibbett S.S. (2009). Multiplex Lateral-Flow Test Strips Fabricated by Two-Dimensional Shaping. ACS Appl. Mater. Interfaces.

[37] Fu E., Lutz B., Kauffman P., Yager P. (2010). Controlled Reagent Transport in Disposable 2D Paper Networks. Lab Chip.

[38] Olkkonen J., Lehtinen K., Erho T. (2010). Flexographically Printed Fluidic Structures in Paper. Anal. Chem..

[39] Dungchai W., Chailapakul O., Henry C.S. (2011). A Low-Cost, Simple, and Rapid Fabrication Method for Paper-Based Microfluidics Using Wax Screen-Printing. Analyst.

[40] Dou M., Sanjay S.T., Benhabib M., Xu F., Li X.J. (2015). Low-Cost Bioanalysis on Paper-Based and Its Hybrid Microfluidic Platforms. Talanta.

[41] Su W., Cook B.S., Fang Y., Tentzeris M.M. (2016). Fully Inkjet-Printed Microfluidics: A Solution to Low-Cost Rapid Three-Dimensional Microfluidics Fabrication with Numerous Electrical and Sensing Applications. Sci. Rep..

[42] Cate D.M., Adkins J.A., Mettakoonpitak J., Henry C.S. (2015). Recent Developments in Paper-Based Micro Fl Uidic Devices. Anal. Chem..

[43] Scida K., Cunningham J.C., Renault C., Richards I., Crooks R.M. (2014). Simple, Sensitive, and Quantitative Electrochemical Detection Method for Paper Analytical Devices. Anal. Chem..

[44] Mahmud M.A., Blondeel E.J.M., Kaddoura M., MacDonald B.D. (2018). Features in Microfluidic Paper-Based Devices Made by Laser Cutting: How Small Can They Be?. Micromachines.

[45] Yu J.H., Jeong S.G., Lee C.S., Hwang J.Y., Kang K.T., Kang H., Lee S.H. (2015). Fabrication of a Paper-Based Analytical Device for Multiple Colorimetric Analysis via Inkjet-Printing and Paper-Cutting. Biochip J..

[46] Liu W., Guo Y., Zhao M., Li H., Zhang Z. (2015). Ring-Oven Washing Technique Integrated Paper-Based Immunodevice for Sensitive Detection of Cancer Biomarker. Anal. Chem..

[47] Jiao Y., Du C., Zong L., Guo X., Han Y., Zhang X., Li L., Zhang C., Ju Q., Liu J. (2020). 3D Vertical-Flow Paper-Based Device for Simultaneous Detection of Multiple Cancer Biomarkers by Fluorescent Immunoassay. Sens. Actuators B Chem..

[48] Li F., Guo L., Hu Y., Li Z., Liu J., He J., Cui H. (2020). Multiplexed Chemiluminescence Determination of Three Acute Myocardial Infarction Biomarkers Based on Microfluidic Paper-Based Immunodevice Dual Amplified by Multifunctionalized Gold Nanoparticles. Talanta.

[49] Yakoh A., Pimpitak U., Rengpipat S., Hirankarn N., Chailapakul O., Chaiyo S. (2021). Paper-Based Electrochemical Biosensor for Diagnosing COVID-19: Detection of SARS-CoV-2 Antibodies and Antigen. Biosens. Bioelectron..

[50] Ge L., Yan J., Song X., Yan M., Ge S., Yu J. (2012). Three-Dimensional Paper-Based Electrochemiluminescence Immunodevice for Multiplexed Measurement of Biomarkers and Point-of-Care Testing. Biomaterials.

[51] Li C., Liu Y., Zhou X., Wang Y. (2020). A Paper-Based SERS Assay for Sensitive Duplex Cytokine Detection towards the Atherosclerosis-Associated Disease Diagnosis. J. Mater. Chem. B.

[52] Hou Y., Lv C.C., Guo Y.L., Ma X.H., Liu W., Jin Y., Li B.X., Yang M., Yao S.Y. (2022). Recent Advances and Applications in Paper-Based Devices for Point-of-Care Testing. J. Anal. Test..

[53] Thomas R. (1961). Colorimetric Detection of Penicillins and Cephalosporins on Paper. Nature.

[54] Bahadır E.B., Sezgintürk M.K. (2016). Lateral Flow Assays: Principles, Designs and Labels. TrAC Trends Anal. Chem..

[55] Calabria D., Calabretta M.M., Zangheri M., Marchegiani E., Trozzi I., Guardigli M., Michelini E., Di Nardo F., Anfossi L., Baggiani C. (2021). Recent Advancements in Enzyme-Based Lateral Flow Immunoassays. Sensors.

[56] Preechakasedkit P., Siangproh W., Khongchareonporn N., Ngamrojanavanich N., Chailapakul O. (2018). Development of an Automated Wax-Printed Paper-Based Lateral Flow Device for Alpha-Fetoprotein Enzyme-Linked Immunosorbent Assay. Biosens. Bioelectron..

[57] Liu C., Gomez F.A., Miao Y., Cui P., Lee W. (2019). A Colorimetric Assay System for Dopamine Using Microfluidic Paper-Based Analytical Devices. Talanta.

[58] Luo Z., Lv T., Zhu K., Li Y., Wang L., Gooding J.J., Liu G., Liu B. (2020). Paper-Based Ratiometric Fluorescence Analytical Devices towards Point-of-Care Testing of Human Serum Albumin. Angew. Chem. Int. Ed..

[59] Fu X., Sompol P., Brandon J.A., Norris C.M., Wilkop T., Johnson L.A., Richards C.I. (2020). In Vivo Single-Molecule Detection of Nanoparticles for Multiphoton Fluorescence Correlation Spectroscopy to Quantify Cerebral Blood Flow. Nano Lett..

[60] Capelo J.L., Roig A., Montalti M., Caponetti V., Trzcinski J.W., Cantelli A., Tavano R., Papini E., Mancin F. (2019). Self-Assembled Biocompatible Fluorescent Nanoparticles for Bioimaging. Front. Chem..

[61] Kim D., Jeong K., Kwon J.E., Park H., Lee S., Kim S., Park S.Y. (2019). Dual-Color Fluorescent Nanoparticles Showing Perfect Color-Specific Photoswitching for Bioimaging and Super-Resolution Microscopy. Nat. Commun..

[62] Chandra A., Prasad S., Gigli G., del Mercato L.L. (2020). Fluorescent Nanoparticles for Sensing. Front. Nanosci..

[63] De Arquer F.P.G., Talapin D.V., Klimov V.I., Arakawa Y., Bayer M., Sargent E.H. (2021). Semiconductor Quantum Dots: Technological Progress and Future Challenges. Science.

[64] Mahmoudi T., Pourhassan-Moghaddam M., Shirdel B., Baradaran B., Morales-Narváez E., Golmohammadi H. (2021). (Nano)Tag-Antibody Conjugates in Rapid Tests. J. Mater. Chem. B.

[65] Chung S., Revia R.A., Zhang M. (2021). Graphene Quantum Dots and Their Applications in Bioimaging, Biosensing, and Therapy. Adv. Mater..

[66] Li X., Tong X., Yue S., Liu C., Channa A.I., You Y., Wang R., Long Z., Zhang Z., Zhao Z. (2021). Rational Design of Colloidal AgGaS2/CdSeS Core/Shell Quantum Dots for Solar Energy Conversion and Light Detection. Nano Energy.

[67] Broughton J.P., Deng X., Yu G., Fasching C.L., Servellita V., Singh J., Miao X., Streithorst J.A., Granados A., Sotomayor-Gonzalez A. (2020). CRISPR–Cas12-Based Detection of SARS-CoV-2. Nat. Biotechnol..

[68] Dodeigne C., Thunus L., Lejeune R. (2000). Chemiluminescence as a Diagnostic Tool. A Review. Talanta.

[69] Sha R., Badhulika S. (2020). Recent Advancements in Fabrication of Nanomaterial Based Biosensors for Diagnosis of Ovarian Cancer: A Comprehensive Review. Microchim. Acta.

[70] Al Mughairy B., Al-Lawati H.A.J. (2020). Recent Analytical Advancements in Microfluidics Using Chemiluminescence Detection Systems for Food Analysis. TrAC Trends Anal. Chem..

[71] Mesgari F., Beigi S.M., Fakhri N., Hosseini M., Aghazadeh M., Ganjali M.R. (2020). Paper-Based Chemiluminescence and Colorimetric Detection of Cytochrome c by Cobalt Hydroxide Decorated Mesoporous Carbon. Microchem. J..

[72] Chen Y.Y., Ting I.J., Wang S.C. (2021). Using Office Inkjet Printer to Develop Paper-Based Electrowetting-on-Dielectric Micromixer Based on Capillary Wave-Induced Droplet Vibration Mixing for the Reproducibility Improvement of Chemiluminescence Assays. J. Taiwan Inst. Chem. Eng..

[73] Xu X., Li H., Hasan D., Ruoff R.S., Wang A.X., Fan D.L. (2013). Near-Field Enhanced Plasmonic-Magnetic Bifunctional Nanotubes for Single Cell Bioanalysis. Adv. Funct. Mater..

[74] Le Ru E.C., Blackie E., Meyer M., Etchegoint P.G. (2007). Surface Enhanced Raman Scattering Enhancement Factors: A Comprehensive Study. J. Phys. Chem. C.

[75] Andersen N.I., Artyushkova K., Matanović I., Seow Chavez M., Hickey D.P., Abdelloui S., Minteer S.D., Atanassov P. (2019). Modular Microfluidic Paper-Based Devices for Multi-Modal Cascade Catalysis. ChemElectroChem.

[76] Fang W., Jia S., Chao J., Wang L., Duan X., Liu H., Li Q., Zuo X., Wang L., Wang L. (2019). Quantizing Single-Molecule Surface-Enhanced Raman Scattering with DNA Origami Metamolecules. Sci. Adv..

[77] Siebe H.S., Chen Q., Li X., Xu Y., Browne W.R., Bell S.E.J. (2021). Filter Paper Based SERS Substrate for the Direct Detection of Analytes in Complex Matrices. Analyst.

[78] Economou A., Kokkinos C., Prodromidis M. (2018). Flexible Plastic, Paper and Textile Lab-on-a Chip Platforms for Electrochemical Biosensing. Lab Chip.

[79] Baharfar M., Rahbar M., Tajik M., Liu G. (2020). Engineering Strategies for Enhancing the Performance of Electrochemical Paper-Based Analytical Devices. Biosens. Bioelectron..

[80] Zanut A., Fiorani A., Canola S., Saito T., Ziebart N., Rapino S., Rebeccani S., Barbon A., Irie T., Josel H.P. (2020). Insights into the Mechanism of Coreactant Electrochemiluminescence Facilitating Enhanced Bioanalytical Performance. Nat. Commun..

[81] Zhao W., Chen H.-Y., Xu J.-J. (2021). Electrogenerated Chemiluminescence Detection of Single Entities. Chem. Sci..

[82] Zhu L., Lv X., Li Z., Shi H., Zhang Y., Zhang L., Yu J. (2021). All-Sealed Paper-Based Electrochemiluminescence Platform for on-Site Determination of Lead Ions. Biosens. Bioelectron..

[83] Ahmadi A., Khoshfetrat S.M., Kabiri S., Dorraji P.S., Larijani B., Omidfar K. (2021). Electrochemiluminescence Paper-Based Screen-Printed Electrode for HbA1c Detection Using Two-Dimensional Zirconium Metal-Organic Framework/Fe3O4 Nanosheet Composites Decorated with Au Nanoclusters. Microchim. Acta.

[84] Sun X., Wan J., Qian K. (2017). Designed Microdevices for In Vitro Diagnostics. Small Methods.

[85] Luppa P.B., Müller C., Schlichtiger A., Schlebusch H. (2011). Point-of-Care Testing (POCT): Current Techniques and Future Perspectives. TrAC Trends Anal. Chem..

[86] Zhang B., Chen M., Cao J., Liang Y., Tu T., Hu J., Li T., Cai Y., Li S., Liu B. (2021). An Integrated Electrochemical POCT Platform for Ultrasensitive CircRNA Detection towards Hepatocellular Carcinoma Diagnosis. Biosens. Bioelectron..

[87] Tian T., Bi Y., Xu X., Zhu Z., Yang C. (2018). Integrated Paper-Based Microfluidic Devices for Point-of-Care Testing. Anal. Methods.

[88] Anderson S., Hadwen B., Brown C. (2021). Thin-Film-Transistor Digital Microfluidics for High Value in Vitro Diagnostics at the Point of Need. Lab Chip.

[89] Li H., Steckl A.J. (2019). Paper Microfluidics for Point-of-Care Blood-Based Analysis and Diagnostics. Anal. Chem..

[90] Bloom D.E., Cadarette D. (2019). Infectious Disease Threats in the Twenty-First Century: Strengthening the Global Response. Front. Immunol..

[91] Excler J.L., Saville M., Berkley S., Kim J.H. (2021). Vaccine Development for Emerging Infectious Diseases. Nat. Med..

[92] Ingle D.J., Howden B.P., Duchene S. (2021). Development of Phylodynamic Methods for Bacterial Pathogens. Trends Microbiol..

[93] Wang C., Liu M., Wang Z., Li S., Deng Y., He N. (2021). Point-of-Care Diagnostics for Infectious Diseases: From Methods to Devices. Nano Today.

[94] Jain S., Nehra M., Kumar R., Dilbaghi N., Hu T.Y., Kumar S., Kaushik A., Li C. (2021). zhong Internet of Medical Things (IoMT)-Integrated Biosensors for Point-of-Care Testing of Infectious Diseases. Biosens. Bioelectron..

[95] Raybould M.I.J., Kovaltsuk A., Marks C., Deane C.M. (2021). CoV-AbDab: The Coronavirus Antibody Database. Bioinformatics.

[96] Gaebler C., Wang Z., Lorenzi J.C.C., Muecksch F., Finkin S., Tokuyama M., Cho A., Jankovic M., Schaefer-Babajew D., Oliveira T.Y. (2021). Evolution of Antibody Immunity to SARS-CoV-2. Nature.

[97] Sheridan C. (2020). Fast, Portable Tests Come Online to Curb Coronavirus Pandemic. Nat. Biotechnol..

[98] Mateos M., Teruel J.L., Nash R., Fernández Lucas M. (1991). Diagnosis of Hepatitis C Virus Infection. Enferm. Infecc. Microbiol. Clin..

[99] Reboud J., Xu G., Garrett A., Adriko M., Yang Z., Tukahebwa E.M., Rowell C., Cooper J.M. (2019). Paper-Based Microfluidics for DNA Diagnostics of Malaria in Low Resource Underserved Rural Communities. Proc. Natl. Acad. Sci. USA.

[100] Witkowska McConnell W., Davis C., Sabir S.R., Garrett A., Bradley-Stewart A., Jajesniak P., Reboud J., Xu G., Yang Z., Gunson R. (2021). Paper Microfluidic Implementation of Loop Mediated Isothermal Amplification for Early Diagnosis of Hepatitis C Virus. Nat. Commun..

[101] Seok Y., Batule B.S., Kim M.G. (2020). Lab-on-Paper for All-in-One Molecular Diagnostics (LAMDA) of Zika, Dengue, and Chikungunya Virus from Human Serum. Biosens. Bioelectron..

[102] Kevadiya B.D., Machhi J., Herskovitz J., Oleynikov M.D., Blomberg W.R., Bajwa N., Soni D., Das S., Hasan M., Patel M. (2021). Diagnostics for SARS-CoV-2 Infections. Nat. Mater..

[103] Wu J., Liu J., Li S., Peng Z., Xiao Z., Wang X., Yan R., Luo J. (2020). Detection and Analysis of Nucleic Acid in Various Biological Samples of COVID-19 Patients. Travel Med. Infect. Dis..

[104] Au W.Y., Peter P., Hang P. (2021). Articles Diagnostic Performances of Common Nucleic Acid Tests for SARS-CoV-2 in Hospitals and Clinics: A Systematic Review and Meta-Analysis. Lancet Microbe.

[105] Harpaldas H., Arumugam S., Rodriguez C.C., Kumar B.A., Shi V., Sia S.K. (2021). Point-of-Care Diagnostics: Recent Developments in a Pandemic Age. Lab Chip.

[106] Song Q., Sun X., Dai Z., Gao Y., Gong X., Zhou B., Wu J., Wen W. (2021). Point-of-Care Testing Detection Methods for COVID-19. Lab Chip.

[107] Soh J.H., Chan H.M., Ying J.Y. (2020). Strategies for Developing Sensitive and Specific Nanoparticle-Based Lateral Flow Assays as Point-of-Care Diagnostic Device. Nano Today.

[108] Deng Y., Jiang H., Li X., Lv X. (2021). Recent Advances in Sensitivity Enhancement for Lateral Flow Assay. Microchim. Acta.

[109] Kasetsirikul S., Umer M., Soda N., Sreejith K.R., Shiddiky M.J.A., Nguyen N.-T. (2020). Detection of the SARS-CoV-2 Humanized Antibody with Paper-Based ELISA. Analyst.

[110] Kasetsirikul S., Umer M., Soda N., Sreejith K.R. (2021). A Paper-Based Immunofluorescent Device for the Detection of SARS-CoV-2 Humanized Antibody. Preprints.

[111] Gong F., Wei H.X., Qi J., Ma H., Liu L., Weng J., Zheng X., Li Q., Zhao D., Fang H. (2021). Pulling-Force Spinning Top for Serum Separation Combined with Paper-Based Microfluidic Devices in COVID-19 ELISA Diagnosis. ACS Sens..

[112] Hristov D., Rijal H., Gomez-Marquez J., Hamad-Schifferli K. (2021). Developing a Paper-Based Antigen Assay to Differentiate between Coronaviruses and SARS-CoV-2 Spike Variants. Anal. Chem..

[113] Han H., Ma Q., Li C., Liu R., Zhao L., Wang W., Zhang P., Liu X., Gao G., Liu F. (2020). Profiling Serum Cytokines in COVID-19 Patients Reveals IL-6 and IL-10 Are Disease Severity Predictors. Emerg. Microbes Infect..

[114] Adrover-Jaume C., Alba-Patiño A., Clemente A., Santopolo G., Vaquer A., Russell S.M., Barón E., González del Campo M.d.M., Ferrer J.M., Berman-Riu M. (2021). Paper Biosensors for Detecting Elevated IL-6 Levels in Blood and Respiratory Samples from COVID-19 Patients. Sens. Actuators B Chem..

[115] Davidson J.L., Wang J., Maruthamuthu M.K., Dextre A., Pascual-Garrigos A., Mohan S., Putikam S.V.S., Osman F.O.I., McChesney D., Seville J. (2021). A Paper-Based Colorimetric Molecular Test for SARS-CoV-2 in Saliva. Biosens. Bioelectron. X.

[116] Dao Thi V.L., Herbst K., Boerner K., Meurer M., Kremer L.P.M., Kirrmaier D., Freistaedter A., Papagiannidis D., Galmozzi C., Stanifer M.L. (2020). A Colorimetric RT-LAMP Assay and LAMP-Sequencing for Detecting SARS-CoV-2 RNA in Clinical Samples. Sci. Transl. Med..

[117] Yu S., Nimse S.B., Kim J., Song K.S., Kim T. (2020). Development of a Lateral Flow Strip Membrane Assay for Rapid and Sensitive Detection of the SARS-CoV-2. Anal. Chem..

[118] Baud D., Gubler D.J., Schaub B., Lanteri M.C., Musso D. (2017). An Update on Zika Virus Infection. Lancet.

[119] Meagher R.J., Negrete O.A., Van Rompay K.K. (2016). Engineering Paper-Based Sensors for Zika Virus. Trends Mol. Med..

[120] Pardee K., Green A.A., Takahashi M.K., Braff D., Lambert G., Lee J.W., Ferrante T., Ma D., Donghia N., Fan M. (2016). Rapid, Low-Cost Detection of Zika Virus Using Programmable Biomolecular Components. Cell.

[121] Compton J. (1991). Nucleic Acid Sequence-Based Amplification. Nature.

[122] Kaarj K., Akarapipad P., Yoon J.Y. (2018). Simpler, Faster, and Sensitive Zika Virus Assay Using Smartphone Detection of Loop-Mediated Isothermal Amplification on Paper Microfluidic Chips. Sci. Rep..

[123] Draz M.S., Venkataramani M., Lakshminarayanan H., Saygili E., Moazeni M., Vasan A., Li Y., Sun X., Hua S., Yu X.G. (2018). Nanoparticle-Enhanced Electrical Detection of Zika Virus on Paper Microchips. Nanoscale.

[124] Rao S., Hossain T., Mahmoudi T. (2021). 3D Human Liver Organoids: An in Vitro Platform to Investigate HBV Infection, Replication and Liver Tumorigenesis. Cancer Lett..

[125] Chen Y., Wang J., Liu Z., Wang X., Li X., Shan G. (2018). A Simple and Versatile Paper-Based Electrochemiluminescence Biosensing Platform for Hepatitis B Virus Surface Antigen Detection. Biochem. Eng. J..

[126] Zhao L., Sun L., Chu X. (2009). Chemiluminescence Immunoassay. TrAC Trends Anal. Chem..

[127] Shen J., Zhou Y., Fu F., Xu H., Lv J., Xiong Y., Wang A. (2015). Immunochromatographic Assay for Quantitative and Sensitive Detection of Hepatitis B Virus Surface Antigen Using Highly Luminescent Quantum Dot-Beads. Talanta.

[128] Sanjay S.T., Dou M., Sun J., Li X. (2016). A Paper/Polymer Hybrid Microfluidic Microplate for Rapid Quantitative Detection of Multiple Disease Biomarkers. Sci. Rep..

[129] Aydın H.B., Cheema J.A., Ammanath G., Toklucu C., Yucel M., Özenler S., Palaniappan A., Liedberg B., Yildiz U.H. (2020). Pixelated Colorimetric Nucleic Acid Assay. Talanta.

[130] Srisomwat C., Teengam P., Chuaypen N., Tangkijvanich P., Vilaivan T., Chailapakul O. (2020). Pop-up Paper Electrochemical Device for Label-Free Hepatitis B Virus DNA Detection. Sens. Actuators B Chem..

[131] Ditmangklo B., Taechalertpaisarn J., Siriwong K., Vilaivan T. (2019). Clickable Styryl Dyes for Fluorescence Labeling of Pyrrolidinyl PNA Probes for the Detection of Base Mutations in DNA. Org. Biomol. Chem..

[132] Tang R., Yang H., Choi J.R., Gong Y., Hu J., Feng S., Pingguan-Murphy B., Mei Q., Xu F. (2016). Improved Sensitivity of Lateral Flow Assay Using Paper-Based Sample Concentration Technique. Talanta.

[133] Dector A., Galindo-de-la-Rosa J., Amaya-Cruz D.M., Ortíz-Verdín A., Guerra-Balcázar M., Olivares-Ramírez J.M., Arriaga L.G., Ledesma-García J. (2017). Towards Autonomous Lateral Flow Assays: Paper-Based Microfluidic Fuel Cell inside an HIV-Test Using a Blood Sample as Fuel. Int. J. Hydrog. Energy.

[134] Lu Q., Su T., Shang Z., Jin D., Shu Y., Xu Q., Hu X. (2021). Flexible Paper-Based Ni-MOF Composite/AuNPs/CNTs Film Electrode for HIV DNA Detection. Biosens. Bioelectron..

[135] Miller B.S., Bezinge L., Gliddon H.D., Huang D., Dold G., Gray E.R., Heaney J., Dobson P.J., Nastouli E., Morton J.J.L. (2020). Spin-Enhanced Nanodiamond Biosensing for Ultrasensitive Diagnostics. Nature.

[136] Hui Y.Y., Cheng C.-A., Chen O.Y., Chang H.-C. (2016). Bioimaging and Quantum Sensing Using NV Centers in Diamond Nanoparticles. Carbon Nanostruct..

[137] Huang J.Y., Lin H.T., Chen T.H., Chen C.A., Chang H.T., Chen C.F. (2018). Signal Amplified Gold Nanoparticles for Cancer Diagnosis on Paper-Based Analytical Devices. ACS Sens..

[138] Yang X., Xia P., Zhang Y., Lian S., Li H., Zhu G., Wang P. (2020). Photothermal Nano-Antibiotic for Effective Treatment of Multidrug-Resistant Bacterial Infection. ACS Appl. Bio Mater..

[139] Chan G.J., Lee A.C., Baqui A.H., Tan J., Black R.E. (2013). Risk of Early-Onset Neonatal Infection with Maternal Infection or Colonization: A Global Systematic Review and Meta-Analysis. PLoS Med..

[140] Chang A.H., Parsonnet J. (2010). Role of Bacteria in Oncogenesis. Clin. Microbiol. Rev..

[141] Ilhan H., Guven B., Dogan U., Torul H., Evran S., Çetin D., Suludere Z., Saglam N., Boyaci İ.H., Tamer U. (2019). The Coupling of Immunomagnetic Enrichment of Bacteria with Paper-Based Platform. Talanta.

[142] He P.J.W., Katis I.N., Kumar A.J.U., Bryant C.A., Keevil C.W., Somani B.K., Mahobia N., Eason R.W., Sones C.L. (2020). Laser-Patterned Paper-Based Sensors for Rapid Point-of-Care Detection and Antibiotic-Resistance Testing of Bacterial Infections. Biosens. Bioelectron..

[143] Kim H.J., Kwon C., Noh H. (2019). Paper-Based Diagnostic System Facilitating Escherichia Coli Assessments by Duplex Coloration. ACS Sens..

[144] Wang Y.C., Tsai Y.H., Shen C.F., He M.Y., Fu Y.C., Sang C.Y., Lee Y.T., Cheng C.M. (2021). Turntable Paper-Based Device to Detect Escherichia Coli. Micromachines.

[145] Li X., Li X., Guo Y., Liu Y., Mei S., Song X., Li J., Grugbaye A.G., Li J., Xu K. (2019). Development and Assessment of a Paper-Based Enzyme-Linked Immunosorbent Assay for the Colorimetric Diagnosis of Human Brucellosis. Anal. Lett..

[146] Alatraktchi F.A.Z.a., Noori J.S., Tanev G.P., Mortensen J., Dimaki M., Johansen H.K., Madsen J., Molin S., Svendsen W.E. (2018). Paper-Based Sensors for Rapid Detection of Virulence Factor Produced by Pseudomonas Aeruginosa. PLoS ONE.

[147] E Silva R.F., Longo Cesar Paixão T.R., Der Torossian Torres M., de Araujo W.R. (2020). Simple and Inexpensive Electrochemical Paper-Based Analytical Device for Sensitive Detection of Pseudomonas Aeruginosa. Sens. Actuators B Chem..

[148] Abe T., Koi C., Kohi S., Song K.B., Tamura K., Macgregor-Das A., Kitaoka N., Chuidian M., Ford M., Dbouk M. (2020). Gene Variants That Affect Levels of Circulating Tumor Markers Increase Identification of Patients With Pancreatic Cancer. Clin. Gastroenterol. Hepatol..

[149] Li Y., Tian X., Gao L., Jiang X., Fu R., Zhang T., Ren T., Hu P., Wu Y., Zhao P. (2019). Clinical Significance of Circulating Tumor Cells and Tumor Markers in the Diagnosis of Lung Cancer. Cancer Med..

[150] Chen J., Li P., Zhang T., Xu Z., Huang X., Wang R., Du L. (2022). Review on Strategies and Technologies for Exosome Isolation and Purification. Front. Bioeng. Biotechnol..

[151] Hassanzadeh-Barforoushi A., Warkiani M.E., Gallego-Ortega D., Liu G., Barber T. (2020). Capillary-Assisted Microfluidic Biosensing Platform Captures Single Cell Secretion Dynamics in Nanoliter Compartments. Biosens. Bioelectron..

[152] Rao D., Mei K., Yan T., Wang Y., Wu W., Chen Y., Wang J., Zhang Q., Wu S. (2021). Nanomechanical Sensor for Rapid and Ultrasensitive Detection of Tumor Markers in Serum Using Nanobody. Nano Res..

[153] Lan Q., Ren C., Lambert A., Zhang G., Li J., Cheng Q., Hu X., Yang Z. (2020). Platinum Nanoparticle-Decorated Graphene Oxide@Polystyrene Nanospheres for Label-Free Electrochemical Immunosensing of Tumor Markers. ACS Sustain. Chem. Eng..

[154] Qi L., Liu S., Jiang Y., Lin J.M., Yu L., Hu Q. (2020). Simultaneous Detection of Multiple Tumor Markers in Blood by Functional Liquid Crystal Sensors Assisted with Target-Induced Dissociation of Aptamer. Anal. Chem..

[155] Gong P., Sun L., Wang F., Liu X., Yan Z., Wang M., Zhang L., Tian Z., Liu Z., You J. (2019). Highly Fluorescent N-Doped Carbon Dots with Two-Photon Emission for Ultrasensitive Detection of Tumor Marker and Visual Monitor Anticancer Drug Loading and Delivery. Chem. Eng. J..

[156] Zhang P., Zhou X., He M., Shang Y., Tetlow A.L., Godwin A.K., Zeng Y. (2019). Ultrasensitive Detection of Circulating Exosomes with a 3D-Nanopatterned Microfluidic Chip. Nat. Biomed. Eng..

[157] Wang N., Wang J., Zhao X., Chen H., Xu H., Bai L., Wang W., Yang H., Wei D., Yuan B. (2021). Highly Sensitive Electrochemical Immunosensor for the Simultaneous Detection of Multiple Tumor Markers for Signal Amplification. Talanta.

[158] Mahmoudi T., Pirpour Tazehkand A., Pourhassan-Moghaddam M., Alizadeh-Ghodsi M., Ding L., Baradaran B., Razavi Bazaz S., Jin D., Ebrahimi Warkiani M. (2020). PCR-Free Paper-Based Nanobiosensing Platform for Visual Detection of Telomerase Activity via Gold Enhancement. Microchem. J..

[159] Ma Y.-H.V., Middleton K., You L., Sun Y. (2018). A Review of Microfluidic Approaches for Investigating Cancer Extravasation during Metastasis. Microsyst. Nanoeng..

[160] Mahmoudi T., Shirdel B., Mansoori B., Baradaran B. (2020). Dual Sensitivity Enhancement in Gold Nanoparticle-based Lateral Flow Immunoassay for Visual Detection of Carcinoembryonic Antigen. Anal. Sci. Adv..

[161] Yonet-Tanyeri N., Ahlmark B.Z., Little S.R. (2021). Advances in Multiplexed Paper-Based Analytical Devices for Cancer Diagnosis: A Review of Technological Developments. Adv. Mater. Technol..

[162] Mahmoudi T., de la Guardia M., Baradaran B. (2020). Lateral Flow Assays towards Point-of-Care Cancer Detection: A Review of Current Progress and Future Trends. TrAC Trends Anal. Chem..

[163] Mahmoudi T., Pourhassan-Moghaddam M., Shirdel B., Baradaran B., Morales-Narváez E., Golmohammadi H. (2021). On-Site Detection of Carcinoembryonic Antigen in Human Serum. Biosensors.

[164] Prasad K.S., Cao X., Gao N., Jin Q., Sanjay S.T., Henao-Pabon G., Li X.J. (2020). A Low-Cost Nanomaterial-Based Electrochemical Immunosensor on Paper for High-Sensitivity Early Detection of Pancreatic Cancer. Sens. Actuators B Chem..

[165] Prasad K.S., Abugalyon Y., Li C., Xu F., Li X. (2020). A New Method to Amplify Colorimetric Signals of Paper-Based Nanobiosensors for Simple and Sensitive Pancreatic Cancer Biomarker Detection. Analyst.

[166] Chu W., Chen Y., Liu W., Zhao M., Li H. (2017). Paper-Based Chemiluminescence Immunodevice with Temporal Controls of Reagent Transport Technique. Sens. Actuators B Chem..

[167] Wang Y., Luo J., Liu J., Sun S., Xiong Y., Ma Y., Yan S., Yang Y., Yin H., Cai X. (2019). Label-Free Microfluidic Paper-Based Electrochemical Aptasensor for Ultrasensitive and Simultaneous Multiplexed Detection of Cancer Biomarkers. Biosens. Bioelectron..

[168] Cinti S., Cinotti G., Parolo C., Nguyen E.P., Caratelli V., Moscone D., Arduini F., Merkoci A. (2020). Experimental Comparison in Sensing Breast Cancer Mutations by Signal on and Signal off Paper-Based Electroanalytical Strips. Anal. Chem..

